# Tunable single-photon emitters in 2D materials

**DOI:** 10.1515/nanoph-2024-0050

**Published:** 2024-06-03

**Authors:** Yi Yu, In Cheol Seo, Manlin Luo, Kunze Lu, Bongkwon Son, Jian Kwang Tan, Donguk Nam

**Affiliations:** School of Electrical and Electronic Engineering, 54761 Nanyang Technological University, 50 Nanyang Avenue, Singapore 639798, Singapore

**Keywords:** 2D materials, single-photon emitters, wavelength tunability, strain engineering, Stark effect

## Abstract

Single-photon emitters (SPEs) hold the key to many quantum technologies including quantum computing. In particular, developing a scalable array of identical SPEs can play an important role in preparing single photons – crucial resources for computation – at a high rate, allowing to improve the computational capacity. Recently, different types of SPEs have been found in various 2D materials. Towards realizing scalable SPE arrays in 2D materials for quantum computation, it is required to develop tunable SPEs that can produce identical photons by precisely controlling emission properties. Here, we present a brief review of the recent progress on various tuning methods in different 2D materials. Firstly, we discuss the operation principle of different 2D SPEs along with their unique characteristics. Secondly, we introduce various dynamic strain engineering methods for tuning the emission wavelengths in 2D SPEs. We also present several electric field-induced wavelength tuning methods for 2D SPEs. Lastly, we discuss the outlook of dynamically tunable 2D SPEs towards scalable 2D SPE arrays for realizing practical quantum photonics applications.

## 1 Introduction

Single-photon emitters (SPEs) are arguably the most crucial component for many quantum technologies, including quantum communication, quantum information processing, quantum metrology, and quantum lithography [1], [2], [3], [4], [5], [6].

In quantum key distribution (QKD), SPEs make undetected eavesdropping impossible, as it is not feasible to replicate a single photon perfectly within an unknown quantum system [6], [7], [8], [9]. The insensitivity of single photons to environmental perturbations makes them ideal quantum bits (qubits), capable of maintaining coherence over a long distance [6], [10], [11]. Multiple degrees of freedom for qubit encoding [11] as well as photon’s high transmission speed promise superior computational power. In quantum metrology, the ability of single photons to eliminate shot noise allows enhancing measurement sensitivity in circumstances with low photon counts [12], [13]. Furthermore, the utilization of entangled single photons enhances the precision of time measurements [14]. Quantum lithography exploits the reduced quantum mechanical wavelength of higher photon number states to surpass the Rayleigh diffraction limit, thus reducing the minimum feature size [15].

High-quality SPEs in terms of several metrics are set by certain requirements of the practical quantum applications. Among these metrics, purity, brightness, and indistinguishability are considered crucial for many practical applications.

SPEs can be distinguished from classical multiphoton emitters (MPEs) based on the ability to emit a single photon rather than more than one photon given a single pulse excitation. Ideal SPEs should emit only single photons at all times, while practical SPEs cannot avoid multiphoton emission processes. The purity of SPEs describes the probability that the emitter undergoes the single-photon emission process, which can be quantified by the second-order correlation at zero time delay *g* ^2^(0) through Hanbury Brown and Twiss (HBT) measurement. In this measurement, emitted photons are directed to a 50:50 beamsplitter and subsequently sent to two single-photon detectors, which record photon-coincidence events at various time delays between the two detectors [16]. Since a single photon cannot be split at the beamsplitter, an ideal SPE producing only single photons would result in no coincidence events at zero delay, yielding *g* ^2^(0) = 0. Conversely, practical SPEs typically show finite coincidence events, producing *g* ^2^(0) > 0 due to the non-zero possibility for multiphoton emission. An emitter is considered an SPE if *g* ^2^(0) < 0.5.

The brightness of SPEs describes the maximum single photon extraction rate in the unit of Hz. The brightness metric is crucial for high-speed and high-fidelity quantum applications such as quantum key distribution (QKD) [8], [9], [17].

Indistinguishability describes the degree to which two single photons cannot be differentiated from each other. This concept is central to the Hong-Ou-Mandel (HOM) effect, a fundamental quantum interference phenomenon. The HOM effect states that when two indistinguishable single photons meet each other at a 50:50 beamsplitter, quantum interference happens, leading to the vanishing probability of two photons exiting the beamsplitter through different ports [18]. The greater the indistinguishability between the photons, the stronger the quantum interference and, consequently, the lower the probability of the photons exiting separately. The visibility of this decreased probability is known as HOM visibility (V_HOM_), which serves as a quantitative indicator of the indistinguishability of two single photons.

Back in 1977, the first SPE was demonstrated by Kimble et al. in sodium atoms [16]. Since then, SPEs have been found in a large variety of host materials, including molecules [19], mesoscopic quantum wells [20], color centers [21], trapped ions [22], semiconductor quantum dots [23], and nanotubes or nanowires [24], [25]. The recent discovery of single-photon emission in a group of 2D materials, including transition metal dichalcogenides (TMDs) [26], [27], [28], [29], [30], [31], [32], [33], [34] and insulating 2D hexagonal boron nitride (hBN) [35], provides a new SPE platform. The unique features of these 2D materials such as the atomic-layer thickness open unprecedented possibilities to engineer and integrate SPEs. For example, the dry transfer technique available for 2D materials allows easy integration of the 2D SPEs onto on-chip waveguides [36]. The ability to stack numerous types of 2D materials with precise alignment to form stable Van der Waals (vdW) heterostructures offers a powerful tool to tailor the 2D SPE properties [37].

The development was initially limited by the constraints regarding the size and quality of monolayers, particularly evident in TMD materials where monolayers serve as preferred hosts for SPEs. Conventional top–down mechanical exfoliation techniques struggle to produce monolayers exceeding the micrometer scale [38], [39], [40], [41], while bottom–up methods like chemical vapor deposition (CVD) face challenges in ensuring uniformity across the produced monolayers [42]. Recent advancements have successfully addressed these challenges through concerted efforts aimed at obtaining large-area TMD monolayers while preserving their quality. For example, by leveraging the robust adhesion between gold and TMD materials, large-area monolayers reaching up to the centimeter scale were successfully exfoliated from bulk TMD materials, while the material quality was proven to be comparable to monolayers obtained via conventional Scotch tape mechanical exfoliation [38], [39], [40], [41]. Furthermore, wafer-scale TMD monolayers that are homogeneous over the entire films were synthesized through the bottom–up metal–organic chemical vapor deposition (MOCVD) [42].

The performance metrics of 2D SPEs, such as the brightness and purity, have also exhibited rapid advancements [43] approaching the required standards for numerous quantum applications. This can be well attributed to the recent breakthrough in improving the 2D material quality. Notably, refinements in the growth process of TMD materials, particularly through methods like chemical vapor transport (CVT) growth [44] and flux growth [45] or chemical treatments by organic superacid [46], have demonstrated efficacy in eliminating defect-mediated non-radiative (NR) recombination, consequently increasing the quantum yield (QY) of SPEs within TMD monolayers. Encapsulating TMD monolayers with hBN flakes [47] can further improve the quality of TMD monolayers by suppressing the inhomogeneous influence from the external environment, which can lead to a narrow excitonic linewidth approaching the homogeneous limit [48], [49] as well as a decreased SPE emission linewidth [50].

In addition to the advances in improving the 2D SPE properties, significant progress in manipulating light-matter interactions at the nanoscale has contributed substantially to enhancing 2D SPE performance. For example, coupling SPEs with dielectric photonic structures [51], [52], [53] and metallic plasmonic nanocavities [44], [45], [54], has notably increased the radiative recombination rate, and consequently, the QY of 2D SPEs through Purcell and plasmonic enhancements, respectively. Moreover, integrating SPEs within an open cavity has optimized single-photon collection efficiency [55]. Other strategies to further enhance collection efficiency include incorporating a gold mirror beneath the SPEs to reflect the emission upwards [56], and using microspheres to collimate the divergent SPE emission [57].

More breakthroughs have been achieved for 2D SPEs such as the electrically driven SPEs in TMDs [58]. However, demonstrating the indistinguishability of single photons emitted from both the same SPE and distinct SPEs in 2D materials encounters numerous difficulties. Particularly, obtaining indistinguishable single photons from distinct 2D SPEs can be a crucial step towards building a scalable array of 2D SPEs – an important component for many quantum applications such as large-scale quantum computation and high-speed quantum communications.

There are several essential factors affecting the indistinguishability of two photons, among which the most crucial factors are wavelength and polarization [59].

To demonstrate the indistinguishability of single photons emitted from the same 2D SPE, the emitter should be free from any spectral diffusion as well as pure dephasing process [60], [61], [62], [63], [64], [65]. The spectral diffusion caused by the fluctuation of the local electric field shifts the energy level of the SPE emission state from time to time [60], [61], [62], [64], while the pure dephasing process caused by the phonon interaction randomly disturbs the single photon emission process and broadens the emission linewidth [61], [62], [63], [64], [65], [66], [67]. Both processes contribute to the randomness in wavelength, disrupting the indistinguishability between emitted single photons. To demonstrate an SPE is free from these two processes, the SPE should give a stable emission linewidth approaching Fourier-transform (FT) limit which is solely determined by the lifetime of the SPE [61], [68], [69], [70]. 2D SPEs with FT limited linewidth have been demonstrated in hBN through photoluminescence excitation (PLE) measurement by decoupling the SPE from the detrimental pure dephasing process naturally or suppressing the spectral diffusion through electric field application [61], [62], [63]. However, the severe spectral diffusion in those hBN SPEs has hindered the confirmation of indistinguishability [43]. It is proposed that resonant excitation with power well below saturation [67] and proper substrate treatments through surface passivation [71] or applying conductive substrate [72] can further minimize spectral diffusion. SPEs in TMDs, especially WSe_2_, also show the potential to achieve FT limited linewidth with smaller spectral diffusion than hBN SPEs [43]. Very recently, Drawer et al. claimed that they observed the two-photon quantum interference from a single SPE in monolayer WSe_2_ in a HOM measurement with 2 % visibility by coupling the SPE to a tunable open cavity [55]. This work demonstrates the first and marks an important milestone in confirming single-photon indistinguishability from a single 2D SPE.

After confirming the indistinguishability of single photons emitted from a single 2D SPE, the immediate next crucial step is to confirm indistinguishability between single photons emitted from two distinct 2D SPEs. A significant challenge in pursuing this step arises because the wavelength and polarization of single photons emitted from any distinct 2D SPEs could vary significantly, despite identical device fabrication processes. These variations are primarily due to the 2D materials’ band structures being highly sensitive to non-uniform strain and electric field distributions, as well as randomly distributed defects within the 2D materials. Such variability complicates matching the wavelength and polarization of photons between distinct 2D SPEs. Therefore, it is essential to implement a post-fabrication treatment that allows monitoring and dynamic tuning of the desired properties, such as emission wavelength and polarization, to ensure the production of indistinguishable single photons from different 2D SPEs.

Efforts to manipulate the emission polarization of 2D SPEs have been made, mainly through strain engineering. Ridges and gaps of various sizes are employed to engineer the strain profile within WSe_2_ monolayers, aligning the polarization of SPE excitonic states [53], [73], [74]. Additionally, the application of uniaxial tensile strain to hBN flakes has proven effective in rotating the original dipole polarization of SPE defect states to align with the strain orientation [75]. Despite these advancements, achieving systematic control over the polarization of 2D SPEs remains under exploration. In contrast, intensive work in dynamically tuning the emission wavelength of 2D SPEs has been done so far.

Here we will review the recent progress on various methods to dynamically tune the emission wavelength of SPEs in 2D materials. This paper is arranged as follows. Section 2 discusses the operation principle of SPEs in most common 2D materials, hBN and WSe_2_. Section 3 introduces dynamic wavelength tuning in SPEs through various strain engineering methods. Section 4 presents various electric field-induced 2D SPE dynamic wavelength tuning methods. In Section 5, we conclude by discussing the outlook of dynamically tunable 2D SPEs towards scalable 2D SPE arrays which in the end leads to the achievement of practical quantum photonics applications.

## 2 Operation principle and characteristics of SPEs in 2D materials

Over the past few years, SPEs have been reported in 2D materials, among which TMDs and hBN are most promising. Numerous experiments and simulations have been performed to study the origins of the SPEs in TMDs and hBN, suggesting various potential explanations for the operation principle of those SPEs. In this section, we review the operation principles that are widely accepted by the research community. We also discuss unique characteristics of 2D SPEs that can be explained by such operation principles.

### 2.1 SPEs in TMDs

SPEs have been found in many TMDs candidates, including MoS_2_ [26], MoSe_2_ [27], WS_2_ [28], WSe_2_ [29], [30], [31], [32], [33], and MoTe_2_ [34]. Among these candidates, the WSe_2_ stands out due to its largest spin-orbit coupling which leads to the largest exciton binding energy, and the existence of a low-lying long-lifetime dark exciton state which leads to an efficient coupling to the SPE states in the bandgap [76], [77].

Five independent research groups first found that isolated defects in WSe_2_ monolayers can emit single photons at cryogenic temperatures [29], [30], [31], [32], [33]. He et al. and Chakraborty et al. even proposed the origin of these SPEs as the excitons trapped at defects [29], [30]. This hypothesis can be supported by a few experimental observations as follows. Firstly, all SPEs in monolayer WSe_2_ reported thus far show sharp isolated emission lines within the defect broadband below the conduction band [78]. A recent statistics study on emission wavelengths of numerous SPEs in WSe_2_ recreated the shape of the defect broadband spectrum, showing a strong correlation between the SPEs and the defect [79]. Secondly, the SPEs in WSe_2_ show emission saturation behaviors above a certain excitation power [80], [81]. This saturation is very common for the emission from defects because of the finite population of defect states. Thirdly, Parto et al. and Xu et al. applied electron beam lithography (EBL) to vary the defect density in monolayer WSe_2_ transferred onto the nanopillar, and showed a direct correlation between the defect density and the number of SPEs [50], [82].

While the above-mentioned experiments indicate that the origin of the SPEs in WSe_2_ could be defects, several recent papers instead focused on the effect of localized strain on creating deterministic SPEs in WSe_2_. For example, a pre-patterned substrate with an array of nanopillars with different sizes was used to induce strong localized strains in monolayer WSe_2_ transferred onto the nanopillars [50], [51], [83], [84], [85]. This suggests that not only the defects but also the strain plays a crucial role in the SPE formation in monolayer WSe_2_.

**Figure 1:**
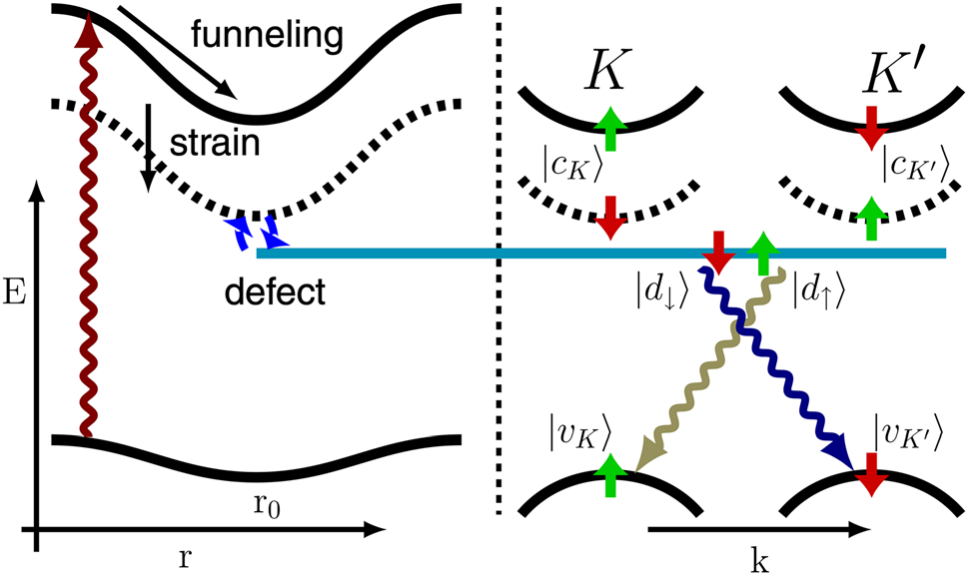
Schematic illustration of single-photon emitter in WSe_2_. Adapted with permission from ref. [86] Copyright 2019, American Physical Society.

Based on the reported experimental features of the WSe_2_ SPEs, Linhart et al. presented a hypothesized model of ‘intervalley defect exciton’ [86]. A localized strain reduces the intervalley dark excitonic and bandgap energies, creating a spatially varying energy band diagram [87], [88], [89], [90]. The quantum well-like potential variation allows the formation of weakly localized dark exciton states [83], which can hybridize with a strongly localized defect state when the energy and wave function of these two states align [86]. This hybridization between the intervalley dark exciton and valley symmetry-breaking defects breaks the valley selectivity (originally the intervalley transition is forbidden [91]), enabling the intervalley dark excitons to radiatively recombine efficiently through the defect states and produce sharp SPE emission lines [86] (Figure 1).

This ‘intervalley defect exciton’ model explains most of the following unique features of SPEs in the monolayer WSe_2_, especially the magnetic response of the SPE doublet emissions. At zero magnetic field, the WSe_2_ SPE shows cross-linearly polarized doublet emission with a splitting of 0.2–1.0 meV, which is because the exchange interaction between excitons leading to a superposition of the cross-circularly polarized emissions from defect states to the valence band in K and K′ valleys [29], [86]. With increasing magnetic field, the doublets gain larger splitting energy, due to the opposite magnetic response of the valence bands in K and K′ valleys [92], which in total contributes to an effective g factor of around 8.8 [29], [86]. In addition, the cross-circularly polarized emissions gradually recover with increasing magnetic fields which is assigned to the large magnetic field strength overwhelming the exchange interaction at high magnetic fields [29], [86]. Recently, Dang et al. successfully explained different *g* factor values from 0 to 12 observed in distinct SPEs in monolayer WSe_2_ by investigating the contributions from various magnetic moments related to conduction and valence band orbitals as well as electrons or holes trapped in the defects [81].

Knowing that the defect states are crucial to the WSe_2_ SPE, it is reasonable to ask what the origins of the defects are in the WSe_2_. The defect origins of the WSe_2_ SPEs have been extensively studied. Zhang et al. first applied low-temperature scanning tunneling microscopy (STM) and scanning tunneling spectroscopy (STS) to CVD monolayer WSe_2_ grown on highly oriented pyrolytic graphite (HOPG) along with ab initio calculations to unveil the prevalent intrinsic point defect origin in the WSe_2_ is the single tungsten vacancy (*V*_*W*_), which can well explain why the monolayer WSe_2_ is p-type doped rather than n-type doped [93]. These intrinsic *V*_*W*_ defects lead to the common defect broadband emission 40–100 meV below the bright exciton states. Most SPEs created on monolayer WSe_2_ through nanopillars fall in these energy ranges, indicating that the intrinsic defects participate in those SPE emissions. Zheng et al. argued that instead of *V*_*W*_, the oxygen interstitial (O_ins_) is the intrinsic defect origin of the SPEs in monolayer WSe_2_ through STM and STEM measurement on a CVD monolayer WSe_2_ grown on sapphire, and first-principles calculations [94]. While the intrinsic defect origin is still under debate, the origin of the EBL-induced extrinsic defect in the monolayer WSe_2_ is proven to be Selenide vacancy (*V*_Se_) with emission energy around 150 meV below the bright exciton [95], [96]. The SPEs created through electron beam treatment, usually fall in this energy range, lower than the SPEs originated from the intrinsic defects [50], [82]. Other defects created by Ar^+^ plasma give defect broadband emission (∼350 meV below bright exciton) much lower than the intrinsic defects and EBL-induced defects [96]. However, this defect origin is still unknown and no SPEs have been observed in WSe_2_ falls in this energy range so far.

### 2.2 hBN SPEs

Contrary to the long debate on the origin of the WSe_2_ SPEs, emission lines from the hBN SPEs have been explained relatively well by localized defect centers deep in the bandgap [35], [60], [97]–[108]. As an insulator, hBN has a large bandgap of ∼6 eV, within which various optically active defect states exist. These defect states produce sharp zero-phonon line (ZPL) emissions in the range from the near-infrared (NIR) to the ultraviolet (UV) regime. These ZPLs emissions are characterized as single photons that are stable both at cryogenic and room temperatures, making the hBN SPEs a promising candidate for practical quantum applications at ambient conditions. The operation principle of the hBN SPEs is the same as the much-studied SPEs in diamond. While diamond has a well-documented library of the SPE-related defects including nitrogen-vacancy (NV), silicon-vacancy (SiV), and germanium-vacancy (GeV) centers, the identification of the defects in hBN that produce specific single-photon emission lines is still under intensive investigation.

Tran et al. first observed robust SPEs with the ZPL at 623 nm (1.99 eV) from both monolayer and multilayer hBN at room temperature, where they assigned the SPE origin to N_B_V_N_ defect [35]. Xu et al. reported a new type of hBN SPEs with a V_B_O_2_ defect emitting at 718 nm (1.73 eV) by employing Ar plasma etching and annealing in an oxygen environment [97]. Gottscholl et al. associated the intrinsic defect V_B_^−^ with the SPEs centered at 850 nm (1.46 eV) after rigorous electron paramagnetic resonance (EPR) measurements [98]. Mendelson et al. presented SPEs producing a 585 nm (2.12 eV) emission line, which shows a direct experimental correlation to the carbon impurity density in the hBN, making V_B_C_N_^−^ defect a potential origin [99]. Bourrellier et al. found bright UV SPEs emitting at 302 nm (4.1 eV) [100]. While this work explained a C_N_ defect as an origin, several research groups later proposed that a C_B_C_N_ defect is the origin of this UV SPE based on first-principles simulations [101], [102], [103], [104]. Very recently, Gale et al. observed the emergence of blue SPEs emitting at 436 nm (2.8 eV) after electron beam irradiation (EBI) followed by thermal annealing [105]. They observed a high correlation between the above-mentioned UV SPEs [100] and the blue SPEs and argued that the EBI separates the C_B_C_N_ defects into isolated C_B_ and C_N_ defects, which are the potential origins of the blue SPEs. In a later work by Zhigulin et al., a C^2^_N_ defect, where a nitrogen site is replaced by a pair of carbon atoms, was pointed as an origin based on the point-group symmetry of the defect state [60].

Various research groups dedicated their efforts to explaining the origins of the experimentally reported hBN SPEs by using theoretical simulations. Two groups conducted density-function theory (DFT) calculations to examine the theoretical lineshape of the emission from a range of hBN point defects and appointed that C_B_V_N_ is a potential SPE source based on the matching of the calculated lineshape with the experimental measured SPE emission lineshape at 1.95 eV [106], [107]. Using similar first-principles calculations, Turiansky et al. argued that the boron dangling bonds are strong candidates for the hBN SPEs around 2.0 eV [108]. Notably, the proposed dangling bond origin can explain why most hBN SPEs appear at the edges, which is due to the large dangling bond density at the edges. This dangling bond proposal can also explain why the SPEs are observed with slightly different emission wavelengths, which is due to the high sensitivity of the dangling bonds to the environment [108].

While the exact origin of a large group of hBN SPEs at different energies is still under debate, it is obvious that the large number of optically active defect states in the hBN offers a promising route for creating SPEs with a wide range of emission energies by carefully controlling the types of defects during the device fabrication.

## 3 Strain engineering for tunable 2D SPEs

As discussed earlier, for building indistinguishable 2D SPE arrays, the ability to dynamically tune the emission wavelengths of 2D SPEs is crucial, since the original emission wavelengths of distinct SPEs are inherently different. Like in other solid-state systems, applying external strain emerges as a promising method to manipulate the optical properties of SPEs hosted in 2D materials.

For instance, for hBN, strain-induced displacements in the lattice sites can effectively tune the SPE emission wavelength by altering the molecular orbitals and perturbing the defect energy levels, since the emission wavelength is purely determined by the energy level of the atom-like defect state [109]. Conversely, the impact of strain for SPEs in WSe_2_ is more complicated because the SPE emission process involves both intervalley dark exciton states and defect states. The lattice deformation in WSe_2_ caused by strain affects the dark exciton and defect states differently [110]. Such modifications in energy states not only adjust the SPE emission wavelength but also the hybridization strength between these states, depending on their energy differences.

The effect of strain for controlling the SPE emission wavelength has also been intensively explored both experimentally and theoretically, proving the efficacy of strain engineering in realizing tunable 2D SPEs. As strain engineering can play a significant role in achieving this challenging goal of on-chip indistinguishability, we review various strain engineering methods used to tune the emission wavelengths of SPEs in TMDs and hBN.

### 3.1 Bending and stretching

Bending and stretching are the simplest methods that can induce tunable strain on 2D materials [111]. 2D materials can be transferred and adhered to a flexible substrate. By bending or stretching the flexible substrate through an external mechanical machine, it is possible to induce large strain on the 2D material. By varying the amount of bending and stretching of the flexible substrate, the level of strain can be dynamically tuned.

**Figure 2:**
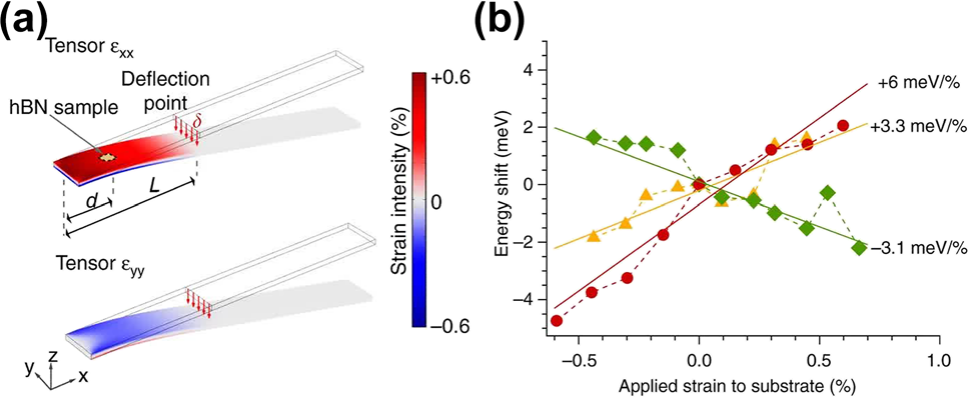
Bending method for dynamic wavelength tuning of hBN SPEs. (a) Schematic of bending the flexible PC beam to apply tunable strain to the hBN SPEs. (b) The energy shift as a function of the applied strain for three hBN SPEs with different tunability from −3.1 meV/% to 6 meV/%. Adapted under the terms of Creative Commons CC BY license [109]. Copyright 2017, Springer Nature.

Grosso et al. first demonstrated the dynamic tuning of the emission wavelength for hBN SPEs through the bending method [109]. A mechanically exfoliated hBN flake was transferred onto a flexible polycarbonate (PC) beam after treating hBN with focused ion beam (FIB) and high-temperature annealing to create SPEs. As illustrated in Figure 2a, one edge of the PC beam was fixed, while the other was bent downward (upward) to apply tensile (compressive) strain to the hBN flake. Based on the amount of bending of the PC beam, it was estimated that tensile and compressive strains of up to 0.6 % can be applied to the hBN flake uniformly.

Figure 2b shows the ZPL emission spectra of two hBN SPEs under different strains. Notably, the two SPEs exhibit different strain tuning rates, which is defined as the emission energy shift per unit strain in the unit of meV/%. The left three spectra for an SPE reveal a blueshift under tensile strain with a strain tuning rate of −3.1 meV/%, while the right three spectra for another SPE show a redshift under tensile strain with a 6 meV/% tuning rate. The variability in strain tuning rates may be attributed to differences in the dipole orientations of defects between the two hBN SPEs. SPE defects with dipole orientation more closely aligned with the uniaxial strain orientation tend to exhibit greater sensitivity to strain-induced changes. The observed discrepancies in the signs of strain tuning rates can be explained by considering the finite Poisson’s ratio of the PC beam which leads to an opposing strain in the orthogonal orientation of the applied strain. When an SPE defect possesses an orthogonal dipole orientation to the applied strain, the SPE emission wavelength undergoes tuning primarily due to the opposing strain rather than the applied strain on the PC beam, resulting in a reversal of the sign of the strain tuning rate.

**Figure 3:**
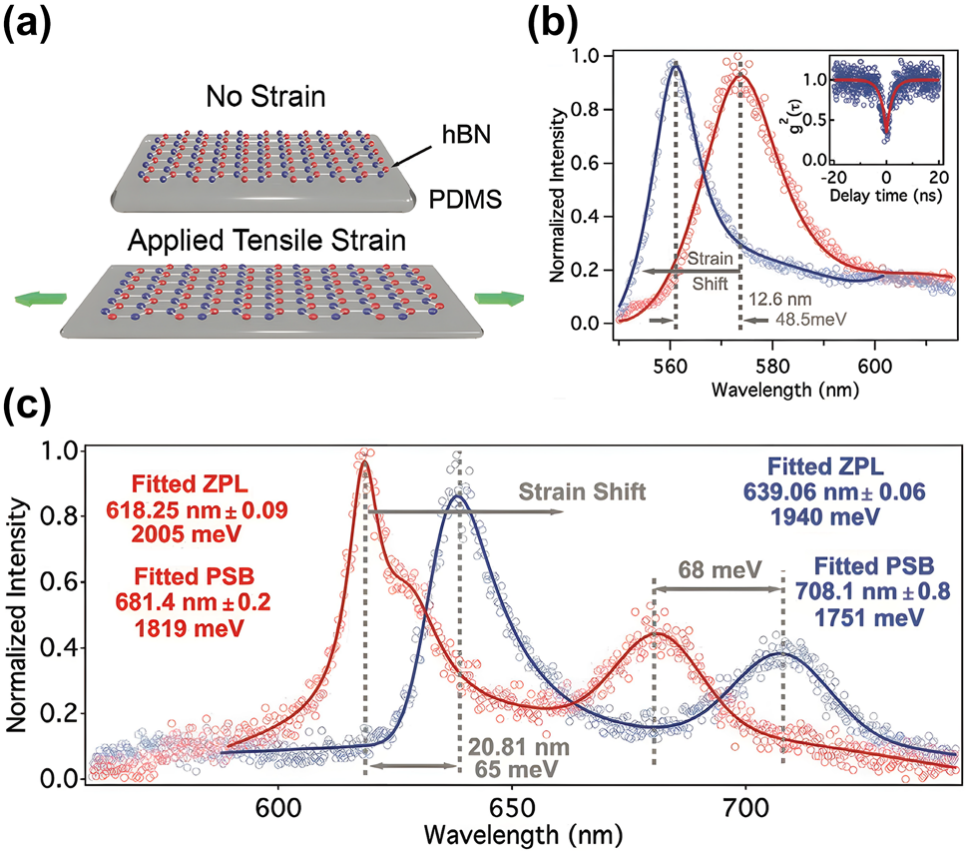
Stretching method for dynamic wavelength tuning of hBN SPEs. (a) Schematic of using mechanical stretching apparatus to apply tunable tensile strain to the hBN SPEs. (b), (c) The spectra when no strain (red) and a tensile strain (blue) is applied to the hBN SPEs with (b) a blue shift and (c) a red shift under tensile strain. Adapted with permission from ref. [75]. Copyright 2020, Wiley-VCH.

Mendelson et al. reported a significantly larger dynamic wavelength tuning range of hBN SPEs by harnessing a stretching method [75]. In their experiment, hBN thin films, grown by chemical vapor deposition (CVD) on copper foil substrates, were wet-transferred onto a PDMS slab, which was secured in a mechanical stretching apparatus (Figure 3a). Unlike previous bending methods, this configuration can only apply tensile strain to the hBN thin films on the PDMS.

For an SPE with ZPL at 573 nm, possibly corresponding to the C_B_V_N_ defect, a blue shift of 48.5 meV was observed for a 3.7 % tensile strain, representing a strain tuning rate of 13.2 meV/% (Figure 3b). Conversely, an opposite redshift of 65 meV was also achieved under a 5.55 % tensile strain for another SPE emitting at 618 nm, possibly associated with the N_B_V_N_ defect, exhibiting a strain tuning rate of −11.7 meV/% (Figure 3c). It was highlighted that the defect dipoles in both SPEs are well aligned with the strain direction, which suggests that the orientation of the defect dipole and the strain field does not appear to be the root cause of the observed opposite signs in strain tuning rates.

To precisely understand the effect of strain on tuning the SPE emission wavelength, it is crucial to quantitatively verify the amount of strain induced in the 2D material. The calculated strain in the flexible substrates under bending and stretching likely exceeds the actual strain applied to the hBN SPEs due to multiple factors. Primarily, the discrepancy arises from the difference in mechanical stiffness. For example, hBN has a significantly larger Young’s modulus compared to flexible substrates, resulting in a reduced strain transfer efficiency from flexible substrates to hBN. This was verified by Gant et al. who calculated the strain transfer efficiency from flexible substrates with various Young’s modulus to MoS_2_ with a modulus of 246 GPa [112]. While a relatively high transfer efficiency of 80 % can be achieved for monolayer MoS_2_ on a PC substrate with a Young’s modulus of 2.4 GPa, the same 2D layer on a PDMS substrate with a modulus of <1 MPa exhibits an extremely low strain transfer efficiency close to zero. For hBN flakes with a very large modulus of ∼865 GPa [113], the strain transfer is anticipated to be less efficient compared to that of MoS_2_.

### 3.2 Electrically induced strain tuning

**Figure 4:**
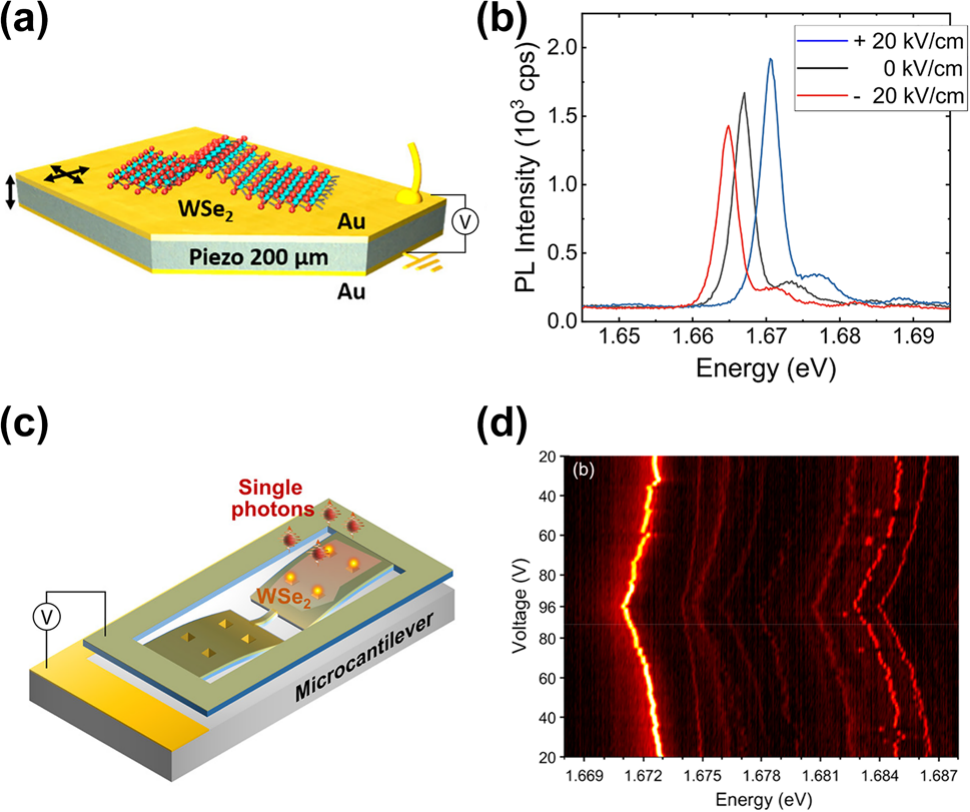
Dynamic wavelength tuning of WSe_2_ SPEs through electrically induced strain. (a) Schematic of applying electrically induced tunable strain to the WSe_2_ SPEs using piezoelectric substrate. (b) The spectra of one WSe_2_ SPE under the strain induced by different voltage biases applied to the piezoelectric substrate from −20 kV/cm to 20 kV/cm. (c) Schematic of the electrically induced strain tuning using Si microcantilever actuated by the electrostatic force. (d) The spectrum map of the WSe_2_ SPEs under strain induced by different voltage biases applied to the microcantilever. Panels (a) and (b): adapted with permission from ref. [114]. Copyright 2019, American Chemical Society. Panels (c) and (d): Adapted with permission from ref. [56]. Copyright 2019, American Chemical Society.

Electrically induced strain tuning methods employing piezoelectric materials can be particularly useful for demonstrating tunable SPEs in TMDs. For example, WSe_2_ SPEs require operational constraints such as cryogenic temperature, preventing the use of bulky mechanical machines for strain tuning. Iff et al. placed monolayer WSe_2_ containing SPEs onto a piezoelectric substrate PMN–PT as illustrated in Figure 4a [114]. An electric field applied perpendicular to the piezoelectric substrates causes expansion or shrinkage of the substrate depending on the polarity of the electric field, allowing to induce tensile or compressive strain into the WSe_2_ layer. Consequently, the emission wavelength of the WSe_2_ SPEs can be controlled in a reversible manner as shown in Figure 4b. A large in-plane bi-axial strain of up to 0.15 % was applied to WSe_2_ SPEs, resulting in a large emission tuning of 18 meV. Different SPEs in a single layer exhibited varying emission tuning rates both in magnitude and sign, which was attributed to non-uniform strain alteration depending on the locations of SPEs.

Another approach for electrically induced strain engineering is the application of microelectromechanical systems (MEMS) techniques, which offers the advantage of CMOS compatibility, unlike piezoelectric substrates. Leveraging electrostatic forces, this method enables the voltage-controlled emission tuning of SPEs. Kim et al. utilized the MEMS technique, where monolayer WSe_2_ was transferred onto Si microcantilevers with nanopyramid arrays, which can deterministically create SPEs via localized strain (Figure 4c) [56]. By varying the voltage applied between the Si cantilevers and an underlying Si substrate, it is possible to control the magnitude of the electrostatic forces that can pull down the Si cantilevers and exert tensile strain on the WSe_2_ layer. As illustrated in Figure 4d, the application of voltages of up to 96 V induced a maximum strain of ∼0.07 %, which resulted in an energy shift of 3.5 meV.

### 3.3 Pressure for strain tuning

The variation of pressure surrounding 2D SPEs is also a convenient approach to achieving dynamic strain tuning. An isotropic hydrostatic pressure in a cryogenic chamber induces both an intralayer in-plane and an interlayer out-of-plane uniform compressive strain on multi-layered 2D materials. Varying the pressure can tune the magnitude of the strain applied to the 2D materials. Xue et al. demonstrated the pressure-induced dynamic tuning of the emission wavelength in hBN SPEs [115].

An anomalous pressure dependence of the SPE emission wavelength was observed, where some SPEs first underwent redshift with pressure, followed by blueshift as the pressure was further increased. Simulations for the N_B_V_N_ defect in hBN revealed that at a low-pressure range, interlayer compressive strain dominates and causes redshift, while at a high-pressure range, intralayer compressive strain overwhelms the interlayer lattice distortion, leading to blueshift. The application of a maximum pressure of up to 4 GPa resulted in an energy shift of 42 meV with a tuning rate of up to 15 meV/GPa.

### 3.4 Surface acoustic wave for strain tuning

**Figure 5:**
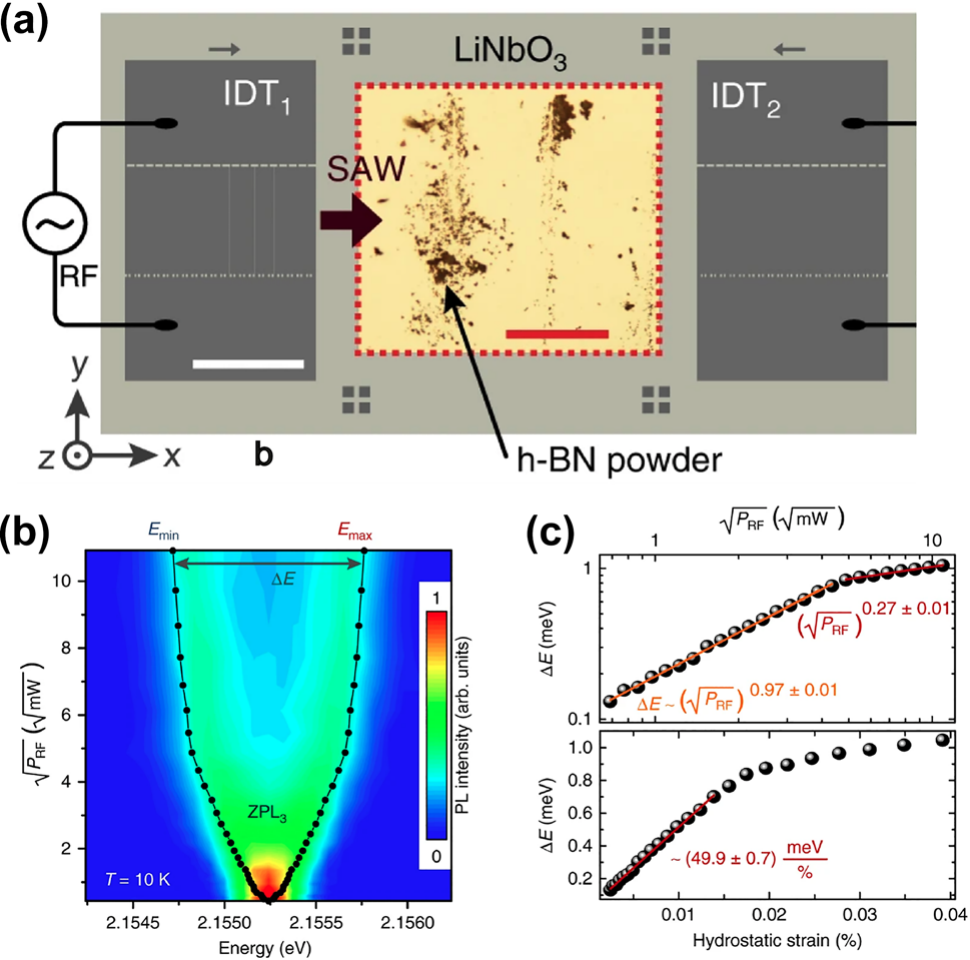
Dynamic wavelength tuning of hBN SPEs through surface acoustic wave. (a) Schematic of the SAW device for inducing periodic strain field to the SPEs in hBN nanoflakes. (b) The false-color spectrum of the SPE emission in hBN nanoflakes under different SAW amplitudes ( $\sqrt{P_{RF}}$ ). (c) The energy splitting (Δ*E*) as a function of the SAW amplitude (up) and the SAW-induced hydrostatic strain to the hBN nanoflakes (down). Adapted under the terms of Creative Commons CC BY license [116]. Copyright 2019, Springer Nature.

Applying surface acoustic wave (SAW) is another well-studied approach to achieve dynamic strain engineering on 2D SPEs. Launching the SAW to the region with 2D materials induces the oscillation of the surface lattice structure, leading to a periodic strain field in the 2D materials. Varying the magnitude of the SAW can tune the strain applied to the 2D materials, leading to dynamic SPE emission tuning. Lazić et al. and Likawa et al. utilized the interdigital transducer (IDT) on the piezoelectric substrate as the SAW source to demonstrate dynamic tuning of the SPE emission wavelength in hBN nanoflakes (Figure 5a) [116], [117]. By tuning the amplitude of the radiative frequency (RF) signal applied to the IDT, a splitting of the hBN SPE emission energy (Δ*E*) was achieved as shown in Figure 5b. After calculating the hydrostatic strain induced by the SAW to the hBN nanoflakes, a maximum SPE emission tuning range of 2.5 meV and strain tuning rate of 40–50 meV/% was deduced (Figure 5c). The strain tuning rate of the SAW method is much higher than that of bending and stretching methods, suggesting a stronger physical contact between the hBN nanoflakes and the vibrating substrate [116].

## 4 Electric field engineering for tunable 2D SPEs

**Figure 6:**
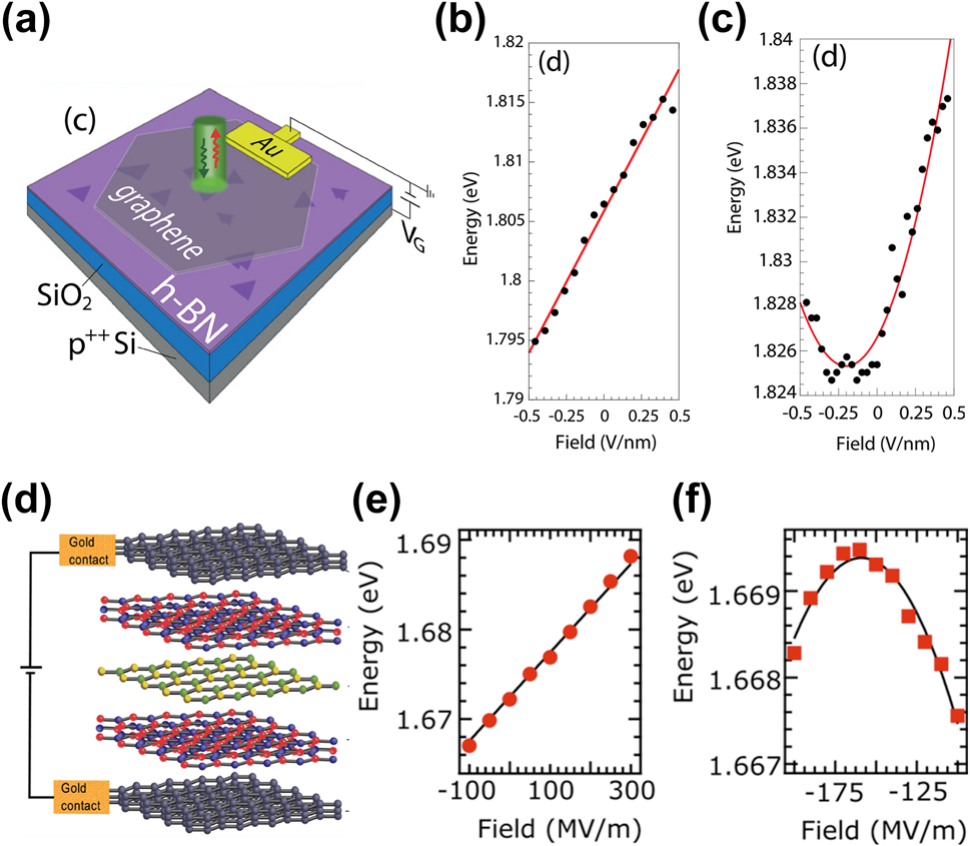
Dynamic wavelength tuning of hBN and WSe_2_ SPEs through out-of-plane electric field application. (a) Schematic of the graphene/hBN/SiO_2_/Si device for applying out-of-plane electric fields to hBN. (b), (c) The emission energy as a (b) linear or (c) quadratic function of the applied electric field for different hBN SPEs. (d) Schematic of the vdW heterostructure device for applying out-of-plane electric fields to WSe_2_ monolayers. The layers from top to bottom are graphene, hBN, WSe_2_ monolayer, hBN, and graphene, respectively. (e), (f) The emission energy as a (b) linear or (c) quadratic function of the applied electric field for different WSe_2_ SPEs. Panels (a)–(c): adapted with permission from ref. [120]. Copyright 2019, American Institute of Physics. Panels (d)–(f) Adapted with permission from ref. [121]. Copyright 2017, American Chemical Society.

The application of electric fields to 2D materials hosting SPEs is another method to achieve dynamic tuning of the SPE emission wavelength. The applied electric fields interact with the electric dipole of SPE states, inducing the Stark shift. In a greater detail, the transition energy of an SPE coupled to an external electric field, $\vec{F}$ , shifts according to the equation $\Delta E=-\Delta \vec{\mu }\cdot \vec{F}-\left(\Delta \vec{\alpha }/2\cdot \vec{F}\right)\cdot \vec{F}$ , where Δ*E* is the Stark shift of emission energy, $\Delta \vec{\mu }$ and $\Delta \vec{\alpha }$ are the differences of permanent dipole moments and polarizabilities between ground and excited states, respectively [60], [118], [119]. Depending on whether the $\Delta \vec{\mu }$ or $\Delta \vec{\alpha }$ dominates, the resulting Stark shift has a linear or quadratic relation with the external electric field as illustrated in Figure 6. For example, if $\Delta \vec{\mu }$ _(_ $\Delta \vec{\alpha }$ _)_ dominates, the change in ∆*E* with respect to $\vec{F}$ follows a linear (quadratic) relationship, as described by the equation for ∆*E* above.

To understand the physical picture behind the two cases, we can treat the non-zero $\Delta \vec{\alpha }$ as the linear dependence of the $\Delta \vec{\mu }$ on the external electric field $\vec{F}$ . It is widely accepted that the $\Delta \vec{\mu }$ and $\Delta \vec{\alpha }$ values for SPEs are determined by the geometric structure of the defects. The linear Stark shift of hBN SPEs is typically attributed to the neutral V_N_X_B_-type defects (X = N, C, or O) because their energetically favored asymmetric structure leads to a permanent dipole [118]. In contrast, the quadratic Stark shift of hBN SPEs can be related to defects with non-degenerate excited states, similar to the nitrogen-vacancy center SPEs in diamond [119]. Similar observations are found in WSe_2_ SPEs as well. The linear Stark shift is attributed to the single Se vacancy defect with a broken out-of-plane inversion symmetry, while the quadratic Stark shift is attributed to the double Se vacancy defect with a preserved symmetry [86].

Both in-plane and out-of-plane electric fields have been proven effective. In this section, we review various methods of applying electric fields to hBN and WSe_2_ that realize dynamic SPE emission tuning.

### 4.1 Out-of-plane electric field tuning

Applying out-of-plane electric fields to 2D SPEs requires electrodes above and below the 2D materials. The advancement of the stacking methods allows fabricating such vdW heterostructures with electrodes using multi-layer graphene.

Scavuzzo et al. demonstrated the application of an out-of-plane electric field to the SPEs hosted in hBN layers by applying voltages between graphene at the top and a heavily doped Si at the bottom (Figure 6a) [120]. By applying high voltages of up to 70 V, out-of-plane electric fields of up to ∼0.46 V/nm were imposed onto the hBN SPEs, achieving the maximum SPE emission tuning of 20 meV with a Stark tuning rate of 24 meV/(V/nm) (Figure 6b).

Applying out-of-plane electric fields to TMD SPEs requires a more complicated vdW heterostructure. The graphene layers cannot directly contact the TMD layers, which otherwise causes the quenching of the TMD SPEs. If the TMD layers directly contact the graphene layers, the excited electron–hole pairs in TMD will quickly transfer to the contacting graphene layer and recombine through non-radiative channels due to graphene’s semi-metallic Dirac bands [122]. Therefore, it is necessary to encapsulate the TMD layer with insulating layers such as hBN for isolating the TMD SPEs from the graphene electrodes.

Several groups fabricated such structures shown in Figure 6d that is suitable for applying out-of-plane electric fields to the TMD SPEs and demonstrated the dynamic SPE emission tuning in monolayer WSe_2_ [85], [121], [123]. Among them, Chakraborty et al. reported the largest SPE emission tuning range of 21 meV by varying the electric field from −0.1 to 0.3 V/nm, corresponding to a large Stark tuning rate of 52 meV/(V/nm) (Figure 6e) [121].

### 4.2 In-plane electric field tuning

**Figure 7:**
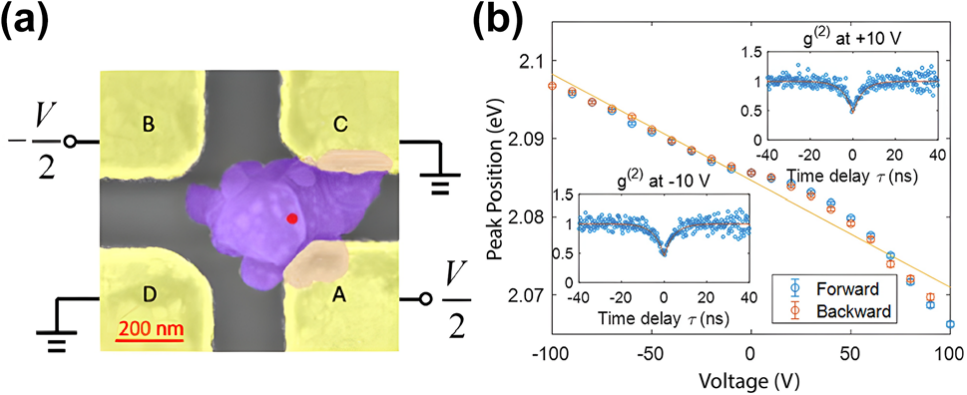
Dynamic wavelength tuning of hBN SPEs through in-plane electric field application. (a) Optical microscope image of the device for applying an in-plane electric field to the hBN SPEs. (b) The spectrum map of the hBN SPE under different in-plane voltage biases from −100 V to 100 V. Adapted with permission from ref. [124]. Copyright 2019, American Chemical Society.

Applying in-plane electric fields to 2D SPEs – especially to those in hBN – is expected to have a stronger Stark effect because most defects in hBN including C_B_V_N_ and N_B_V_N_ have dominant in-plane dipole moments. Xia et al. successfully applied an in-plane electric field to the hBN SPEs by using four in-plane electrodes surrounding the hBN layer as shown in Figure 7a [124]. Having the four in-plane electrodes allows applying an in-plane electric field to the hBN SPE along any direction. By carefully aligning the in-plane electric field along the direction of the dipole moment of the N_B_V_N_ SPE defect, a large SPE emission tuning range of 31 meV was achieved with a Stark tuning rate of 43 meV/(V/nm) (Figure 7b), which is larger than other reported values using out-of-plane electric fields.

### 4.3 Tip-induced local electric field tuning

**Figure 8:**
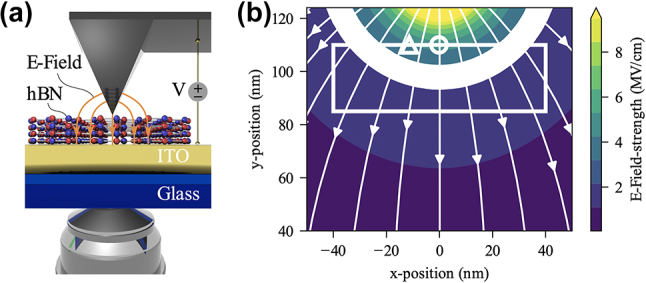
Dynamic wavelength tuning of hBN SPEs through tip-induced electric fields. (a) Schematic of the tip-induced local electric field device. (b) The simulated spatial electric field distribution around the AFM tip when a voltage of 20 V is applied between the AFM tip and the ITO substrate. Adapted with permission from ref. [125]. Copyright 2019, American Physical Society.

It is also possible to apply a very large out-of-plane electric field to SPEs by utilizing a conductive atomic force microscope (AFM) tip (Figure 8a). As shown in Figure 8b, when hBN is placed between the AFM tip and an indium tin oxide (ITO) substrate, dense local electric field lines can be generated, inducing a large electric field across the hBN SPE. An electric field of up to 0.5 V/nm can be locally applied to the hBN SPE with only 20 V, leading to a maximum average SPE emission tuning range of 15.4 meV [125].

## 5 Conclusion and outlook

Since the first report on the SPEs in 2D materials [29]–[33], [35], technological advancement in 2D material growth, defect engineering, and *ab initio* theoretical investigation has been remarkably rapid, suggesting that the development of ideal 2D SPEs as practical quantum sources is imminent. Other advanced techniques manipulating the light-matter interactions through various cavities allow further enhancement of the SPEs by increasing the QY and collection efficiency. One of the critical milestones for realizing a scalable quantum photonics platform with 2D SPEs is to build an array of identical 2D SPEs producing indistinguishable photons. Unlike intrinsically identical ionized atoms used in trapped-ion quantum computers, initially fabricated 2D SPEs are not identical to each other in several crucial factors such as wavelength and polarization, even though the fabrication processes are the same. Therefore, the creation of identical 2D SPEs requires post-fabrication tuning technologies that can precisely control the emission properties of each 2D SPEs individually. In this review, we first discussed the operation principles of SPEs in TMD and hBN. Then, we reviewed several dynamic wavelength tuning methods that are widely used for 2D SPEs including strain and electric field engineering.

While we focused on wavelength control of 2D SPEs, the application of strain and electric fields can also improve other physical properties of 2D SPEs. For example, applying an electric field to the SPEs in monolayer WSe_2_ can improve the purity of emitted single photons through the dissociation of weakly bound background excitons [126]. Applying an electric field to the SPEs in hBN can suppress the spectral diffusion and achieve a superior SPE emission with near-FT limited linewidths, which is pivotal in achieving the indistinguishability of single photons from the same SPE [62]. The use of strain fields demonstrates the ability to control the polarization of SPEs in WSe_2_ monolayers, which is important for achieving identical SPEs [53], [73], [74]. It is also demonstrated that tensile strain can narrow the emission linewidth of hBN SPEs at room temperature [75]. In conclusion, the variety of the 2D SPE tuning methods reviewed in this work offers a powerful toolset and high degrees of freedom for controlling key optical properties of 2D SPEs, paving the way toward achieving arrays of indistinguishable quantum sources for a scalable quantum photonics platform.

Despite significant progress, several challenges must be addressed to realize indistinguishable arrays of SPEs in 2D materials. A major issue with the current methods of tuning SPE emissions is the inability to tune each SPE individually. Most of the techniques discussed in this paper including bending, stretching, and piezoelectric effects affect all SPEs within the same 2D flake simultaneously, prohibiting the wavelength-matching between different SPEs on the same chip. Developing techniques to fine-tune individual SPEs through localized, dynamic strain engineering could revolutionize wavelength-matching strategies, allowing for the realization of multiple indistinguishable SPEs in a single 2D flake.

Another challenge lies in understanding the precise origins of the various SPE defects in terms of atomic structures. There is still a debate on the atomic structures of SPE defects in WSe_2_ [93], [94], while the configuration of most SPE defects in hBN remains unclear. This lack of detailed knowledge about the SPE defect structures hinders the deterministic fabrication of certain SPEs with controlled wavelengths. Furthermore, the sensitivity of the emission wavelength to external strain or electric fields strongly depends on the defect atomic structures [75], [85], [86], [118], [120], [121]. Therefore, a thorough understanding of the defect origins for SPEs could also guide the selection of efficient wavelength tuning methods.

In addition, severe pure dephasing and spectral diffusion caused by electron–phonon interactions and local electric field fluctuations within 2D materials remain the main barrier to demonstrating indistinguishability among single photons emitted from 2D SPEs, although many efforts discussed in this paper are dedicated to understanding and addressing this issue [61], [62], [63], [67], [71], [72]. To eliminate pure dephasing and spectral diffusion in 2D SPEs, it is critical to obtain a more comprehensive understanding of the various SPE defect origins and their susceptibility to decoherence mechanisms in the 2D materials.

**Figure 9:**
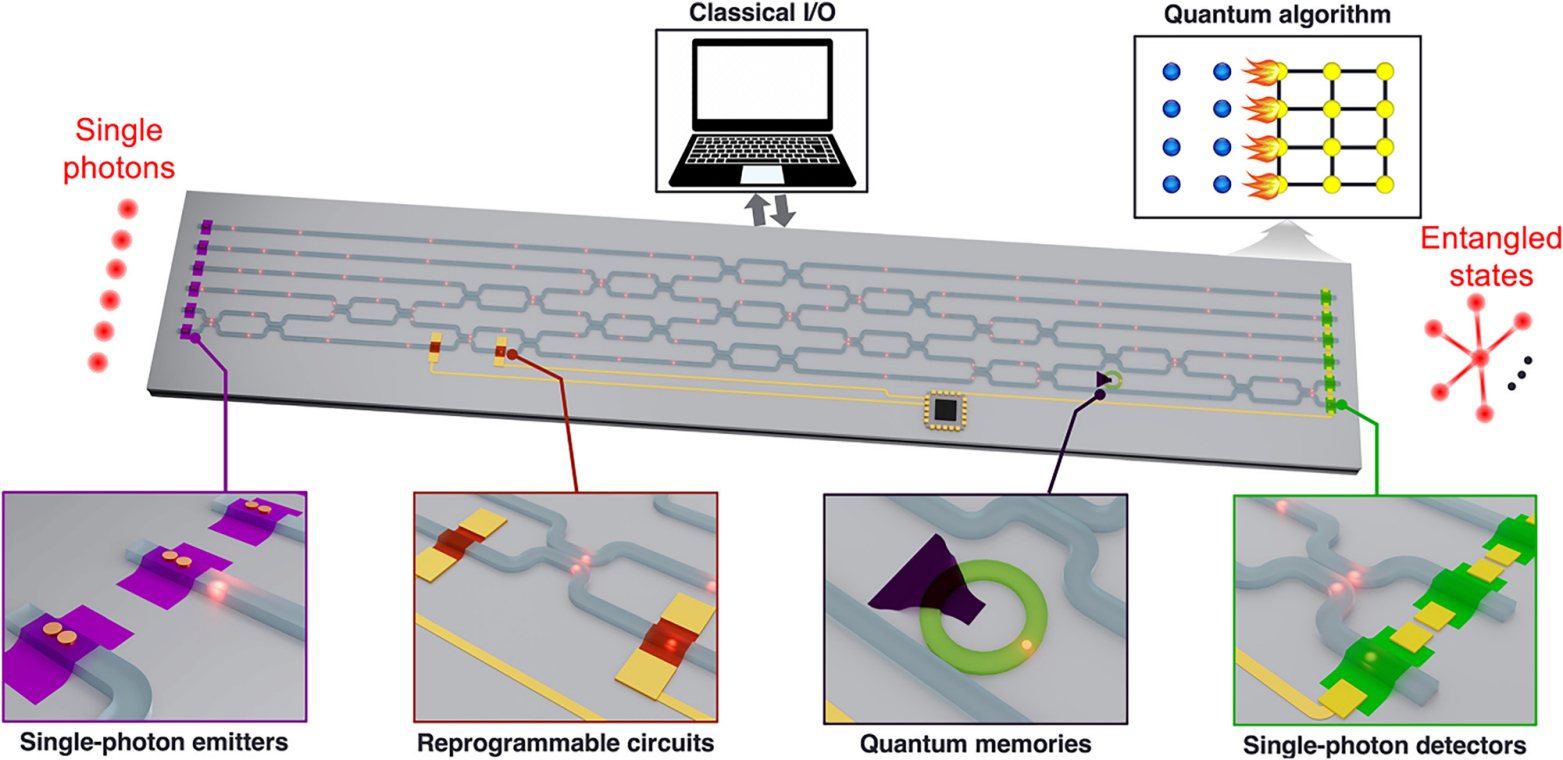
Schematic illustration of an integrated quantum photonic circuit. The bottom panels show four major components: single-photon emitters, reprogrammable circuits, quantum memories, and single-photon detector.

By overcoming the abovementioned challenges, the development of scalable indistinguishable SPE arrays from 2D materials could be within reach. These SPE arrays, when integrated with other crucial quantum photonic components including reprogrammable photonic circuits [127], quantum memories [128], and single-photon detectors [129], may set the stage for a single-chip quantum photonic circuits, as illustrated in Figure 9. By allowing for the manipulation of key parameters of quantum photonic components through external input, such integrated circuits may enable executing diverse quantum algorithms on a scalable platform.
